# Collapsin Response Mediator Protein 2 and Endophilin2 Coordinate Regulation of AMPA Receptor GluA1 Subunit Recycling

**DOI:** 10.3389/fnmol.2020.00128

**Published:** 2020-08-04

**Authors:** Jifeng Zhang, Jiong Li, Yichen Yin, Xueling Li, Yuxin Jiang, Yong Wang, Caihui Cha, Guoqing Guo

**Affiliations:** ^1^Department of Anatomy, Neuroscience Laboratory for Cognitive and Developmental Disorders, Medical College of Jinan University, Guangzhou, China; ^2^Department of Neurology, Guangzhou Red Cross Hospital, Medical College, Jinan University, Guangzhou, China; ^3^Department of Forensic Science, School of Basic Medical Sciences, Central South University, Changsha, China; ^4^Department of Pediatrics, Guangzhou Women and Children’s Medical Center, Guangzhou, China

**Keywords:** collapsing response mediator protein 2, endophilin2, AMPA receptor, GluA1 subunit, recycling

## Abstract

The dynamic trafficking of AMPA receptors (AMPARs), which enables the endocytosis, recycling, and exocytosis of AMPARs, is crucial for synaptic plasticity. Endophilin2, which directly interacts with the GluA1 subunit of AMPARs, plays an important role in AMPAR endocytosis. Collapsin response mediator protein 2 (CRMP2) promotes the maturation of the dendritic spine and can transfer AMPARs to the membrane. Although the mechanisms of AMPAR endocytosis and exocytosis are well known, the exact molecular mechanisms underlying AMPAR recycling remain unclear. Here, we report a unique interaction between CRMP2 and endophilin2. Our results showed that overexpression of CRMP2 and endophilin2 increased the amplitude and frequency of miniature excitatory synaptic currents (mEPSCs) and modestly enhanced AMPAR levels in hippocampal neurons. Furthermore, the CRMP2 and endophilin2 overexpression phenotype failed to occur when the interaction between these two proteins was inhibited. Further analysis revealed that this interaction was regulated by CRMP2 phosphorylation. The phosphorylation of CRMP2 inhibited its interaction with endophilin2; this was mainly affected by the phosphorylation of Thr514 and Ser518 by glycogen synthase kinase (GSK) 3β. CRMP2 phosphorylation increased degradation and inhibited the surface expression of AMPAR GluA1 subunits in cultured hippocampal neurons. However, the dephosphorylation of CRMP2 inhibited degradation and promoted the surface expression of AMPAR GluA1 subunits in cultured hippocampal neurons. Taken together, our data demonstrated that the interaction between CRMP2 and endophilin2 was conductive to the recycling of AMPA receptor GluA1 subunits in hippocampal neurons.

## Introduction

AMPA-type glutamate receptors (AMPARs) are tetrameric assemblies of two dimers that consist of four subunits (GluA1–GluA4); the predominant conformation involves GluA1/GluA2 and GluA2/GluA3 heteromers (Lu et al., 2009; Herguedas et al., 2016). In the central nervous system, AMPARs enable most excitatory transmissions to occur and are crucial for mediating basal synaptic strength and plasticity. Long-term potentiation and long-term depression are the most common forms of synaptic plasticity, which together regulate learning and memory. The dynamic trafficking of AMPARs into and out of synaptic membranes involves the regulation of the number of AMPARs at the synaptic plasma membrane and is crucial for synaptic plasticity (Huganir and Nicoll, 2013). The number of synaptic AMPARs is regulated through endocytosis, exocytosis, and endosomal sorting, which results in the recycling of AMPARs back to the plasma membrane or degradation in the lysosome (van der Sluijs and Hoogenraad, 2011; Parkinson and Hanley, 2018). This dynamic behavior of AMPARs is usually regulated by protein–protein interactions (Bissen et al., 2019).

Endophilin2 is an important regulatory protein involved in clathrin-dependent endocytosis (Chen et al., 2003; Milosevic et al., 2011). High levels of endophilin2 are expressed in the central nervous system, and endophilin2 is enriched in both the pre- and postsynaptic membrane (Chowdhury et al., 2006). Previous studies have shown that endophilin2 can regulate synaptic vesicle endocytosis in the presynaptic membrane as well as receptor endocytosis in the postsynaptic membrane (Chowdhury et al., 2006; Zhang et al., 2015). Moreover, the early onset gene Arc/Arg3.1 interacts with endophilin2/3 and dynamin and enhances receptor endocytosis (Chowdhury et al., 2006). In our previous study, we found that endophilin2 interacted directly with the C-terminus of the GluA1 subunit, but not the GluA2 subunit, to mediate oligomeric amyloid-β-induced AMPAR endocytosis (Zhang et al., 2017). Although the role of endophilin in regulating AMPAR endocytosis is known, the fate of the receptor after undergoing endocytosis by endophilin is unclear. Because AMPARs are subjected to constitutive and regulated exocytosis, endocytosis, and recycling as well as the degradation pathway, studies of the fate of AMPARs after endocytosis by endophilin2 are essential.

Collapsin response mediator proteins (CRMPs), a class of microtubule-associated proteins, are composed of five homologous cytosolic phosphoproteins (CRMP1–5; Minturn et al., 1995; Fukada et al., 2000; Fukata et al., 2002; Yoshimura et al., 2005). CRMP2 was the first protein to be discovered within the CRMP family and is highly expressed in developing and adult nervous systems, particularly in the highly plastic areas of the adult brain, such as the hippocampus, olfactory bulb, and cerebellum (Charrier et al., 2003). At the subcellular level, CRMP2 is normally concentrated at synaptic terminals and in the cytosol of neurons (Yoshimura et al., 2005). Many studies have shown that CRMP2 has roles in cell migration and differentiation, neuronal maturation, neurite extension, and axonal regeneration (Arimura et al., 2000, 2004; Inagaki et al., 2001; Yoshimura et al., 2005; Patrakitkomjorn et al., 2008; Ip et al., 2014). CRMP2 is also required for trafficking N-type voltage-sensitive Ca^2+^ channels and synaptic AMPAR (Brittain et al., 2011; Hensley et al., 2011; Abe et al., 2018). In recent years, many studies have shown that CRMP2 plays an important role in dendritic spine formation and maturation. For example, the density and maturation of the dendritic spine decreased in cortical (Makihara et al., 2016) and hippocampal neurons (Zhang et al., 2016) of CRMP2-knockout mice. The results of our previous study have shown that in neurons overexpressing CRMP2, both the density and dendritic spine maturation were significantly increased (Zhang et al., 2018). Moreover, the PSD95 density, amplitude, and frequency of miniature excitatory synaptic currents (mEPSCs) were also increased (Zhang et al., 2018). The maturation of the dendritic spine indicated an enlargement of the spine head, which provided more area for the insertion of postsynaptic receptors (Zhang et al., 2018). Although some studies have shown that CRMP2 can bind to actin to mediate synaptic AMPAR trafficking (Gu et al., 2010; Hensley et al., 2011; Abe et al., 2018), it is unknown where these receptors come from and how these receptors bridge to the cytoskeletal transport pathway.

In this study, we evaluated and characterized the interactions between CRMP2 and endophilin2. We found that CRMP2 interacted with endophilin2, and their interactions could increase the amplitude and frequency of mEPSCs and the surface expression levels of the AMPAR GluA1 subunit. Furthermore, our data also indicated that the interaction between CRMP2 and endophilin2 was regulated by CRMP2 phosphorylation. Overall, our study provided experimental evidence to support the roles of CRMP2 and endophilin2 in the coordinated regulation of AMPAR recycling.

## Materials and Methods

### Animals

All experiments were conducted with 1-day-old and 1-month-old Sprague–Dawley rats purchased from the Experimental Animal Center of the Sun Yat-sen University (Guangzhou, China). All animal procedures were performed in strict accordance with the recommendations in the Guide for the Care and Use of Laboratory Animals produced by the National Institutes of Health. The protocol was approved by the Institutional Animal Care and Use Committee at Jinan University, China. All efforts were made to minimize the suffering of the animals and to reduce the number of animals used.

### Plasmids

Green fluorescence protein (GFP)-Endo2 and GFP-CRMP2 plasmids were maintained within our laboratory (Zhang et al., 2017, 2018). Full-length CRMP2 and endophilin2 were cloned into PCMV-Tag2B (cat. no. 211172; Agilent Technologies, Santa Clara, CA, USA), pmCherry-C1 (cat. no. 632524, Clontech, Mountain View, CA, USA), and PGEX-5X-3 plasmids (cat. no. 27-4586-01, Clontech, Mountain View, CA, USA). The complementary DNAs (cDNAs) encoding rat CRMP2^ΔC275^ (amino acids 276–572 deleted), CRMP2^ΔC322^ (amino acids 323–572 deleted), CRMP2^ΔC381^ (amino acids 381–562 deleted), CRMP2^ΔN381^ (amino acids 1–380 deleted), Endo2^ΔC121^ (amino acids 122–368 deleted), Endo2^ΔC303^ (amino acids 304–368 deleted), Endo2^122–302^ (amino acids 122–302), and Endo2^ΔN303^ (amino acids 1–302 deleted) were amplified with polymerase chain reaction (PCR) and subcloned into the PGEX-5X-3 plasmid. A two-step PCR method was used to replace the phosphorylation site of CRMP2 with alanine or aspartate. During the first PCR step, complimentary oligo primers were used to generate two DNA fragments with overlapping ends that contained the mutated bases. The overlapping ends were annealed, and the resulting fusion product served as a template for the second PCR. All constructs were verified by sequencing. Ad-HA and Ad-HA-GSK3β S9A [constitutively active glycogen synthase kinase (GSK) 3β mutant containing a serine-to-alanine substitution at residue 9] were purchased from Guangzhou IGE Biotechnology Limited (Guangzhou, China).

### Simulation of the Protein Binding Conformation

The structures of the endophilin2 SH3 domain (PDB code: 3C0C) and CRMP2 (5X1A) were downloaded from the Protein Data Bank^1^. Protein surface electrostatic potential energy was calculated using APBS, and figures were generated using PyMOL (Baker et al., 2001). The initial binding conformation of the CRMP2 and endophilin2 SH3 domain was constructed manually, based on their opposite electrostatic potentials, after which the energy minimization of the protein complex was performed using the molecular dynamics software NAMD v2.13 (Phillips et al., 2005).

### Cell Culture and Transfection

Rat hippocampal neurons were cultured as described previously (Zhang et al., 2012). Calcium phosphate transfections with various constructs were conducted on 10 DIV, and all experiments were carried out on 12 DIV. HEK293 cells (Shanghai Institutes for Biological Sciences) were cultured as described previously (Zhang et al., 2012). Lipofectamine 2000 (Invitrogen, Thermo Fisher Scientific, USA) was used to transfect various constructs into HEK293 cells. Briefly, after replacement of serum-free Dulbecco’s modified Eagle’s medium (DMEM) before transfection, 10 μg plasmid DNA was mixed with 500 μl Optimem (Invitrogen, Thermo Fisher Scientific, USA). Five minutes later, a mixture containing 500 μl Optimem and 30 μl Lipofectamine 2000 was added. After 15–20 min, the mixture was added to a 10-cm culture dish.

### Pharmacological Treatments

Cultured hippocampal neurons were treated for 24 h with dimethyl sulfoxide (DMSO) or 3 μmol CHIR-99021 (Selleckchem, Houston, TX, USA), which was directly added to the neuron culture medium.

### Recombinant Protein Expression and GST Pull-Down Assay

Glutathione-S-transferase (GST)-fusion protein expression and pull-down assays were performed as described previously (Tan et al., 2015). Various GST fusion protein expression vectors were transformed into the BL21 (DE3) strain of *Escherichia coli* (Invitrogen). GST fusion protein was purified using glutathione agarose beads (Pierce Biotechnology, Rockford, IL, USA) according to the manufacturer’s instructions. To ensure that equal amounts of GST and GST fusion proteins were used for the pull-down assay, the samples were stained with Coomassie blue after electrophoresis, and semiquantitative analysis was performed using bovine serum albumin (BSA) as a standard. Then, equal amounts of GST or GST fusion proteins (~10 μg) were mixed with rat brain lysate (~400 μg), and tubes were incubated for 10 h. The samples were centrifuged and analyzed by sodium dodecyl sulfate polyacrylamide gel electrophoresis (SDS-PAGE).

### Immunoprecipitation Assay

HEK293 cells were transfected with GFP, GFP-Endo2 and Flag, and Flag-CRMP2 vectors. After 48 h, cells were centrifugated and lysed in cold radioimmunoprecipitation assay (RIPA) lysis buffer (25 mM Tris–Cl pH 7.4, 100 mM NaCl, 1 mM ethylenediaminetetraacetic acid, 0.5% NP40, and protease inhibitor cocktail). Cell extracts were incubated with protein A/G agarose for 30 min and quantified using bicinchoninic acid (BCA) assays, after solubilization in 500 μl (1 μg/ml) of lysis buffer. Then, cell extracts were immunoprecipitated with 4 μg anti-Flag or 1 μg anti-GFP antibodies and incubated with 30 μl protein A/G agarose overnight at 4°C. The immune complexes were centrifuged and washed five times with wash buffer. The precipitated complexes were collected, and Western blotting analysis was performed.

### Western Blotting

Western blot analysis was performed as described previously (Tan et al., 2015). Briefly, proteins were separated by SDS-PAGE on 10% gels and transferred onto polyvinylidene difluoride membranes. Membranes were blocked in 5% milk in Tris-buffered saline with Tween 20 (TBST) at room temperature and incubated overnight at 4°C with primary antibodies, including rabbit anti-CRMP2, and rabbit anti-GFP (Abcam, Cambridge, UK), mouse anti-endophilin2, and mouse anti-Flag antibodies (Sigma, USA). After rinsing the membranes 3–5 times with TBST, membranes were incubated with horseradish peroxidase-conjugated secondary antibodies (Jackson ImmunoResearch, West Grove, PA, USA) and visualized using enhanced chemiluminescence reagents.

### Fluorescence Immunostaining

Hippocampal neurons were processed for immunofluorescence analysis, according to a previously described standard protocol (Zhang et al., 2017). After transfection for 24–48 h, hippocampal neurons were fixed with 4% paraformaldehyde (Sigma, USA) and 4% sucrose (Sigma, USA) for 15 min at room temperature. Cells were permeabilized with 0.1% (*v*/*v*) Triton X-100 dissolved in TBS for 15 min, and blocked in 3% (*w*/*v*) BSA in TBS for 1 h at room temperature. The cells were then incubated overnight with primary antibodies in blocking buffer at 4°C. Primary antibodies targeting CRMP2 (1:100; Cell Signaling Technology, Danvers, MA, USA) and endophilin2 (E-15; 1:100; Santa Cruz Biotechnology, Dallas, TX, USA) were used for immunocytochemistry. The cells were then washed three times with TBST and incubated with the secondary antibodies Dylight 488 and Dylight 555 (1:1,000; Life Technologies, USA). As per the protocol for staining the surface GluA1, living cells were washed with the extracellular solution for 5 min and blocked in an extracellular solution containing 0.5% BSA for 1 h. The cells were then incubated with primary anti-GluA1 antibodies (1:150; Millipore, Burlington, MA, USA) in blocking buffer for 1.5 h at 4°C. Cells were fixed with 4% paraformaldehyde (Sigma, USA) containing 4% sucrose (Sigma, USA) for 10 min at room temperature. After washing twice, hippocampal neurons were incubated with Dylight 647 (Life Technologies, Carlsbad, CA, USA) in phosphate-buffered saline (PBS) at a 1:1,000 dilution for 1 h at room temperature. After three washes, cells were mounted on glass slides using Fluoro-Gel II without DAPI (Electron Microscopy Sciences, Hatfield, PA, USA).

For AMPAR internalization assay, live hippocampal neurons were labeled with anti-GluA1 antibodies (1:150; Millipore, USA) by incubating coverslips in conditioned medium for 10 min at 37°C. After briefly washing the neurons in prewarmed DMEM, cells were stimulated for 2 min with 50 μM *N*-methyl-D-aspartate (NMDA), returned to conditioned medium and incubated for 5 min. Neurons were fixed with 4% paraformaldehyde (Sigma, USA) containing 4% sucrose (Sigma, USA) for 10 min at room temperature. Receptors remaining on the surface were incubated with Dylight 488 (1:1,000; Life Technologies, Carlsbad, CA, USA) secondary antibodies (not shown in the figure). Internalized receptors were detected with Dylight 647 (Life Technologies, Carlsbad, CA, USA) secondary antibodies after permeabilizing cells in 0.1% (*v*/*v*) Triton X-100 for 15 min. To determine the destiny of internalized receptors, neurons were immunostained with antibodies against early endosome antigen 1 (EEA1; 1:100; Cell Technology, Carlsbad, CA, USA) and lysosomal-associated membrane protein 1 (LAMP1; 1:100; Abcam, USA) overnight at 4°C. The cells were then washed three times with TBST and incubated with Dylight 647 (1:1,000; Life Technologies, Carlsbad, CA, USA) secondary antibodies for 1 h at room temperature. After immunofluorescence staining was performed, results were visualized using a Carl Zeiss LSM 700 confocal microscope (Zeiss, Germany). Fluorescence-integrated density measurements were made in ImageJ software (version ImageJ 2.x; NIH), and Pearson’s correlation coefficients were generated using ImageJ Coloc 2 plugin (Analyze-Colocalization-Coloc 2).

### Electrophysiology

Electrophysiology assays were performed as previously described (Zhang et al., 2017). Whole-cell recordings of mEPSCs were performed at room temperature (20–22°C) from the transfected cultured hippocampal neurons on DIV 11–13. The patch electrode intracellular solution contained the following: 147 mM KCl, 5 mM Na_2_-phosphocreatine, 2 mM ethylene glycol tetraacetic acid (EGTA), 10 mM HEPES, 2 mM MgATP, and 0.3 mM Na_2_GTP; the osmolarity was between 280 and 290 mosmol^−1^. The bathing extracellular solution was of the following composition: 128 mM NaCl, 5 mM KCl, 2 mM CaCl_2_, 1 mM MgCl_2_, 15 mM glucose, 20 mM HEPES, 1 mM tetrodotoxin (TTX), and 0.1 mM picrotoxin (PTX); the osmolarity was between 310 and 320 mosmol^−1^. Recordings were performed at room temperature in voltage clamp mode, at a holding potential of −70 mV, using a Multiclamp 700B amplifier (Molecular Devices, Sunnyvale, CA, USA) and Clampex 10.5 software (Axon Instruments, Foster City, CA, USA). The series resistance was below 30 MΩ, and data were acquired at 10 kHz and filtered at 1 kHz. mEPSCs were analyzed using MiniAnalysis software (Synaptosoft, Inc., Decatur, GA, USA).

### Statistical Analysis

Data are presented as means ± standard errors of the means. The statistical significance of the differences between two groups was analyzed using Student’s *t*-tests and one-way analysis of variance, followed by the Bonferroni or Tamhane *post hoc* tests for comparisons among more than two groups. Results with *P* < 0.05 were considered statistically significant.

## Results

### CRMP2 and Endophilin2 Interacted With Each Other

**Figure 1:**
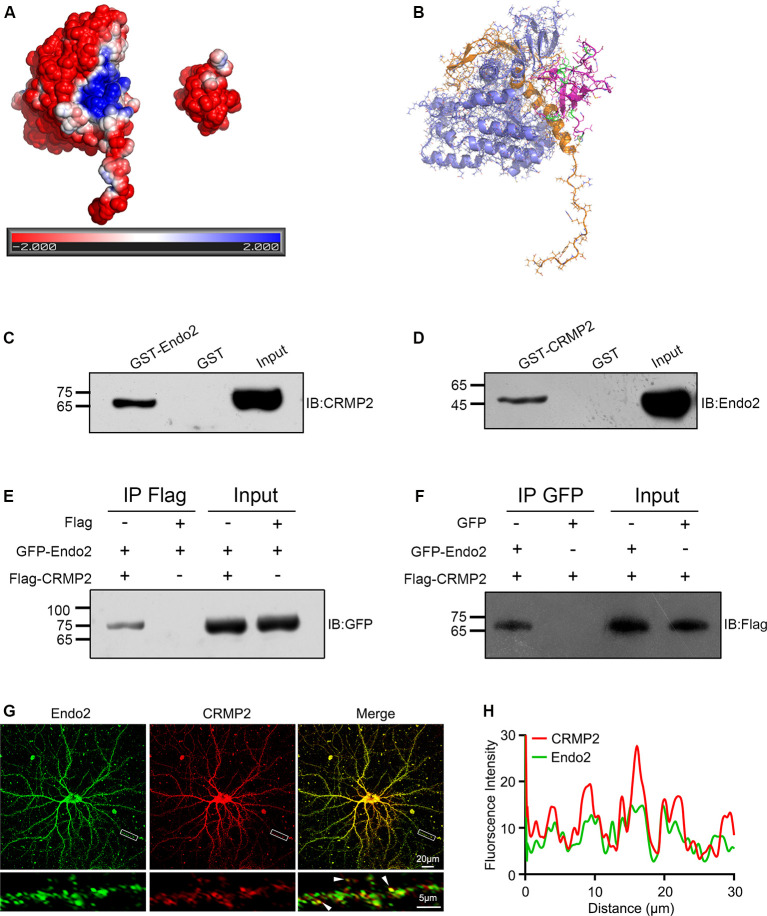
Collapsin response mediator protein 2 (CRMP2) interacts with endophilin2. **(A)** Electrostatic surface potential maps of CRMP2 (left) and endophilin2 SH3 domain (right). The blue color represents the positive potential, and red color represents the negative potential. The electrostatic potential energy of the protein surface is calculated by APBS (figures are produced by using PyMOL). **(B)** The structures of the SH3 domain of endophilin2 (PDB code: 3C0C) and CRMP2 (5X1A) were downloaded from the Protein Data Bank www.rcsb.org. **(C)** Purified glutathione-S-transferase (GST) and GST-Endo2 proteins were incubated with 1-month-old Sprague–Dawley rat brain lysates. Input and bound proteins were analyzed by immunoblotting, using antibodies against CRMP2. **(D)** Purified GST and GST-CRMP2 proteins were incubated with 1-month-old Sprague–Dawley rat brain lysates. Input and bound proteins were analyzed by immunoblotting, using antibodies against Endo2. **(E,F)** HEK293 cells were cotransfected with green fluorescence protein (GFP)-Endo2 and Flag-CRMP2, followed by the co-IP assay. Protein complexes coeluted with the anti-Flag antibody were detected using the anti-GFP antibody **(E)** and protein complexes coeluted with the anti-GFP antibody were detected using the anti-Flag antibody **(F)**. All experiments were repeated independently at least three times. **(G)** Representative image of DIV12 cultured hippocampal neurons stained for endophilin2 and CRMP2. White arrowheads indicate endophilin2 puncta that colocalized with CRMP2. Scale bars of 20 and 5 μm in the magnified dendrite. **(H)** Intensity trace of the endophilin2 and CRMP2 along the dendrite.

To investigate the relationships between CRMP2 and endophilin2, we first searched the STRING protein interaction database; no interactions between these two proteins were reported. Then, we manually modeled the potential binding conformation of CRMP2 and endophilin2 based on their electrostatic potential surface, as calculated by APBS, followed by energy minimization using NAMD v2.13 (Phillips et al., 2005). CRMP2 had a negative electrostatic potential on most of its surface, and there was a cavity with a positive electrostatic potential at the interface of its C-terminal domain. The SH3 domain of endophilin2, which could be properly docked to the cavity close to the CRMP2 C-terminal domain interface, exhibited a negative electrostatic potential (Figures 1A,B). These results suggested that CRMP2 might be able to bind to endophilin2 through an electrostatic interaction. To test these predictions, we constructed GST-CRMP2 and GST-endophilin2 (GST-Endo2) plasmids and purified the proteins. Brain lysates of 1-month-old rats were incubated with GST, GST-CRMP2, and GST-Endo2 fusion proteins, for GST pull-down assays. The results showed that GST-Endo2 interacted with CRMP2 (Figure 1C) and that GST-CRMP2 interacted with endophilin2 (Figure 1D). Moreover, coimmunoprecipitation experiments showed that CRMP2 and endophilin2 could be precipitated with each other (Figures 1E,F). These results indicated that CRMP2 could physically interact with endophilin2 *in vitro*. In addition, to test whether CRMP2 and endophilin2 could interact with each other in cells, we performed the immunofluorescence staining using hippocampal neurons on DIV 12. As shown in Figure 1G, endogenous endophilin2 (green) and CRMP2 (red) were expressed in whole neurons, and their distribution was granule-like. In merged images, yellow granules were observed in cell bodies and neurites, and the fluorescence intensity trace of endophilin2 was consistent with that of CRMP2 (Figure 1H), suggesting colocalization of CRMP2 and endophilin2. The above-mentioned results indicated that CRMP2 could interact with endophilin2.

**Figure 2:**
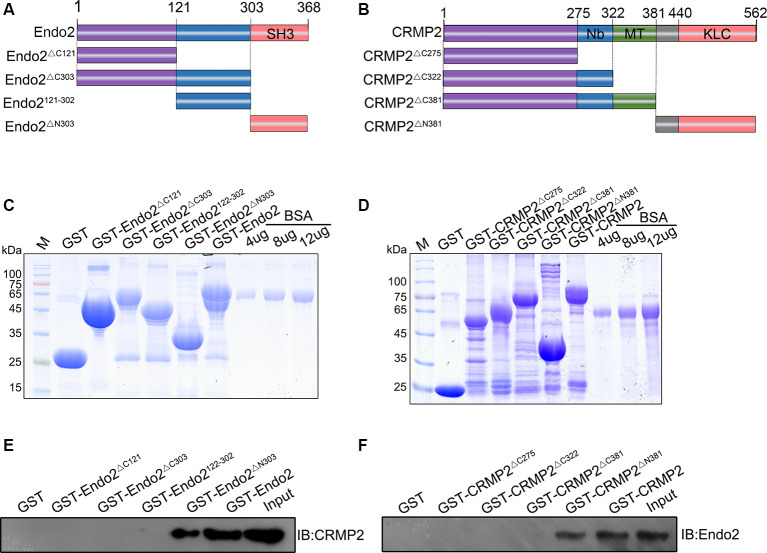
C-terminal residues 303–368 of endophilin2 interact with C-terminal residues 381–562 of CRMP2. **(A,B)** Schematic diagrams of endophilin2 and CRMP2 truncation mutants are shown according to their different domains. **(C)** Purification of glutathione-S-transferase (GST) and truncated segments of GST-endophilin2 (GST-Endo2) fusion proteins. Bovine serum albumin (BSA) standard protein: 4, 8, and 12 μg. **(D)** Purification of GST and truncated segments of GST-CRMP2 fusion proteins. BSA standard protein: 4, 8, and 12 μg. **(E)** Pull-down assays were conducted with GST-Endo2 and its truncation mutants using 1-month-old Sprague–Dawley rat brain lysates. Western blotting was performed with an anti-CRMP2 antibody. **(F)** Pull-down assays were conducted with GST-CRMP2 and its truncation mutants using 1-month-old Sprague–Dawley rat brain lysates. Western blotting was performed with an anti-endophilin2 antibody. All experiments were repeated independently at least three times.

Next, we wanted to locate the specific binding sites of these two proteins. Thus, different types of truncated segments were constructed, according to the schematic diagrams for endophilin2 and CRMP2 (Figures 2A,B). The purified truncated segments of GST-Endo2 and GST-CRMP2 fusion proteins (Figures 2C,D) were used in GST pull-down assays. CRMP2 was detected in GST-Endo2 and GST-Endo2^ΔN303^ sediments that contained the SH3 domain (Figure 2E), and endophilin2 was detected in GST-CRMP2 and GST-CRMP2^ΔN381^ sediments (Figure 2F). This indicated that the SH3 domain of endophilin2 interacted with the C-terminus of CRMP2. These results were consistent with the predicted results in Figures 1A,B.

### CRMP2 and Endophilin2 Coordinated to Enhance Synaptic AMPAR Levels in Hippocampal Neurons

**Figure 3:**
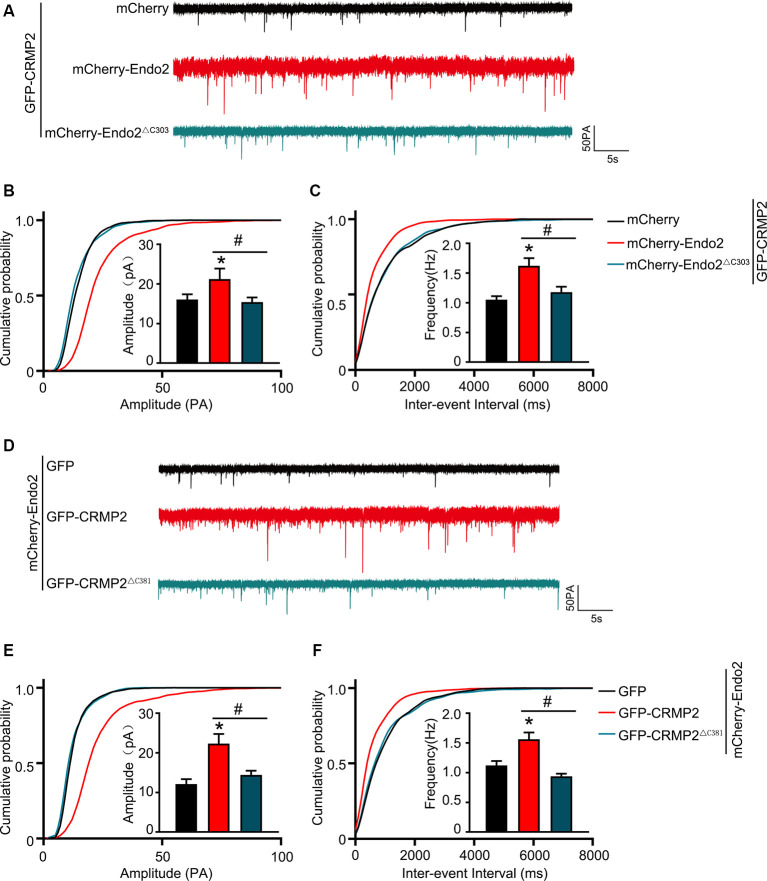
CRMP2 and endophilin2 coordinate with each other to promote the current level of AMPA receptors (AMPARs). **(A)** Representative tracings of miniature excitatory synaptic current (mEPSC) recorded from cultured hippocampal neurons transfected with mCherry-Endo2 and GFP, mCherry-Endo2 and GFP-CRMP2, and mCherry-Endo2 and GFP-CRMP2^ΔC381^. **(B,C)** Cumulative probabilities of the mEPSC amplitude and interevent interval of neurons in these groups. Insets: histogram plots of mEPSC amplitude and frequency of neurons in these groups, *n* = 15 cells. *p < 0.05, as compared to the mCherry-Endo2 and GFP group; ^#^*p* < 0.05, as compared to the mCherry-Endo2 and GFP-CRMP2 group. **(D)** Representative tracings of mEPSC recorded from cultured hippocampal neurons transfected GFP-CRMP2 and mCherry, GFP-CRMP2 and mCherry-Endo2, and GFP-CRMP2 and mCherry-Endo2^ΔC303^. **(E,F)** Cumulative probabilities of the mEPSC amplitude and interevent interval of neurons in these groups. Insets: Histogram plots of mEPSC amplitude and frequency of neurons in these groups, *n* = 15 cells. *p < 0.05, as compared to the GFP-CRMP2 and mCherry group, ^#^*p* < 0.05, as compared to the GFP-CRMP2 and mCherry-Endo2 group.

As mentioned in Introduction, both CRMP2 and endophilin2 performance affect AMPAR trafficking (Chowdhury et al., 2006; Hensley et al., 2011; Zhang et al., 2017; Abe et al., 2018). Moreover, our results showed that CRMP2 interacted with endophilin2; hence, we hypothesized that the interaction between CRMP2 and endophilin2 may influence synaptic AMPAR levels. To test this hypothesis, CRMP2 and endophilin2, together with their truncated mutants, were cotransfected into DIV 12 hippocampal neurons. Then, 48 h after transfection, mEPSCs were recorded using whole-cell patch clamp recordings in order to measure the function of synaptic AMPA receptors (AMPARs). As shown in Figures 3A–C, significant increases in the mEPSC amplitude and frequency were observed in neurons cotransfected with endophilin2 and CRMP2, compared with those from neurons transfected with endophilin2 alone. In contrast, neurons cotransfected with Endo2^ΔC303^ and CRMP2 did not exhibit these enhanced effects. The mEPSC amplitude and frequency were also increased simultaneously in neurons cotransfected with endophilin2 and CRMP2, compared with those from neurons transfected with CRMP2 alone. Neurons cotransfected with CRMP2^ΔC381^ and Endo2 did not exhibit these enhanced effects (Figures 3D–F). These results indicated that CRMP2 and endophilin2 coordinated with each other to promote synaptic AMPAR levels and that inhibition of their interaction could abolish this effect.

**Figure 4:**
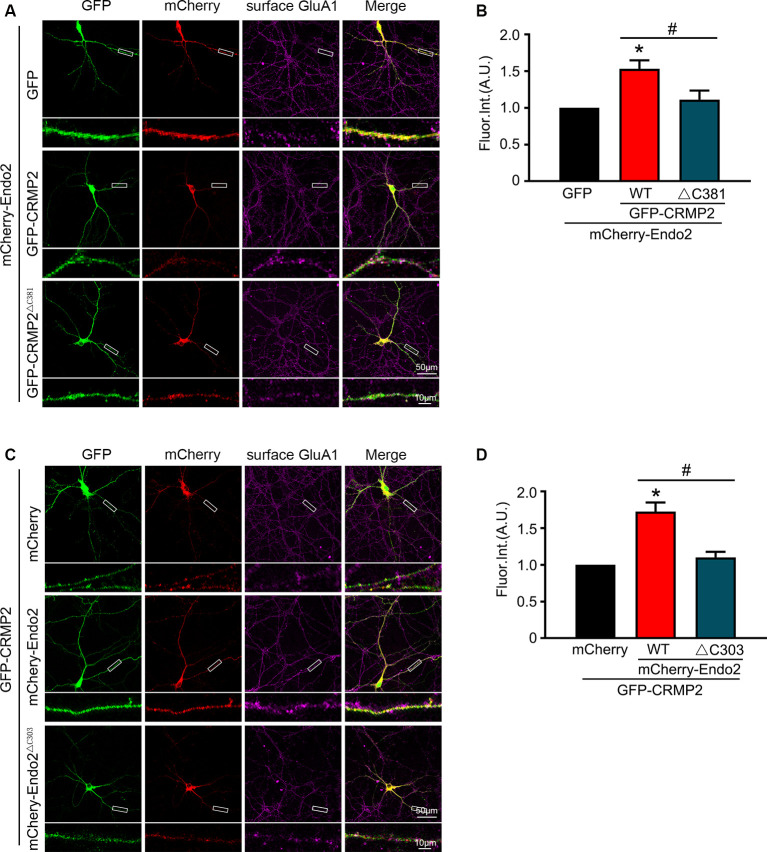
CRMP2 and endophilin2 coordinate to promote the surface expression of AMPA receptor (AMPAR) GluA1 subunit. **(A)** Cultured hippocampal neurons transfected with mCherry-Endo2 and GFP, mCherry-Endo2 and GFP-CRMP2, and mCherry-Endo2 and GFP-CRMP2^ΔC381^ were immunostained with surface GluA1. Scale bar, 50 and 10 μm in the magnified dendrite. **(B)** Quantitative analysis of fluorescence intensity of surface GluA1 of neurons in these groups, *n* = 15 cells per group. *p < 0.05, as compared to the mCherry-Endo2 and GFP group; ^#^*p* < 0.05, as compared to the mCherry-Endo2 and GFP-CRMP2 group. **(C)** Cultured hippocampal neurons transfected with GFP-CRMP2 and mCherry, GFP-CRMP2 and mCherry-Endo2, and GFP-CRMP2 and mCherry-Endo2^ΔC303^ were immunostained with surface GluA1. Scale bar, 50 and 10 μm in the magnified dendrite. **(D)** Quantitative analysis of fluorescence intensity of surface GluA1 of neurons in these groups, *n* = 15 cells per group. *p < 0.05, as compared to the GFP-CRMP2 and mCherry group; ^#^*p* < 0.05, as compared to the mCherry-Endo2 and GFP-CRMP2 group.

In our previous study, we showed that endophilin2 interacted with AMPA receptor GluA1 subunits. To further clarify the effects of the interaction between CRMP2 and endophilin2 on AMPAR levels, we monitored surface AMPAR levels *via* immunocytochemistry analysis of nonpermeabilized neurons with anti-GluA1 antibodies. As shown in Figure 4, the fluorescence intensity of surface GluA1 in neurons cotransfected with endophilin2 and CRMP2 was significantly higher than that in neurons transfected with endophilin2 or CRMP2 alone. If neurons were cotransfected with Endo2^ΔC303^ and CRMP2 or cotransfected with CRMP2^ΔC381^ and Endo2, these enhanced effects were lost. Thus, these results clearly indicated that CRMP2 and endophilin2 coordinated with each other to increase synaptic AMPA receptor levels in hippocampal neurons.

### Phosphorylation of CRMP2 Decreased Its Interaction With Endophilin2

**Figure 5:**
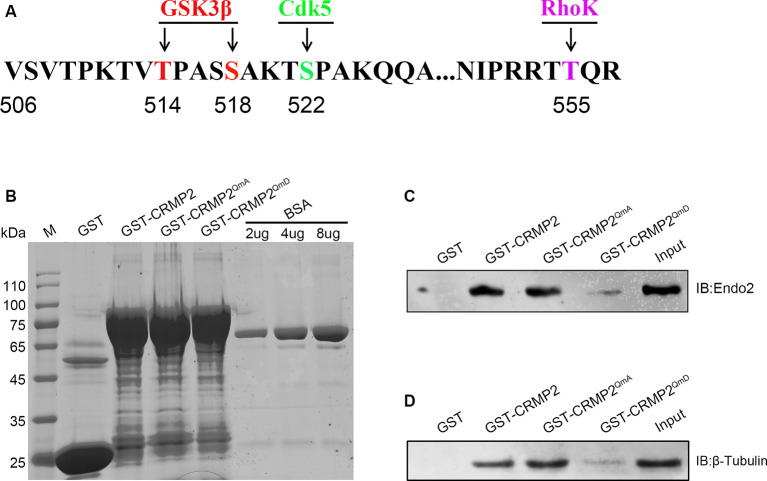
The phosphorylation of CRMP2 decreases its interaction with endophilin2. **(A)** Schematic of GSK3β and Cdk5 and RhoK phosphorylation sites within the rat CRMP2 sequence. Numbers represent amino acid residues within the CRMP2 sequence. **(B)** The expression and purification of glutathione-S-transferase (GST), GST-CRMP2, and its phosphomimetic and dephosphomimetic mutants. BSA standard protein: 2, 4, and 8 μg. **(C)** Pull-down assays were conducted using GST-CRMP2 and its phosphomimetic and dephosphomimetic mutants in brain lysates of 1-month-old Sprague–Dawley rats. Western blotting was performed using an anti-endophilin2 antibody. **(D)** Pull-down assays were conducted with GST-CRMP2 and its phosphomimetic and dephosphomimetic mutants in brain lysates of 1-month-old Sprague–Dawley rats. Western blotting was performed with an anti-tubulin antibody. Each result is representative of three separate experiments with similar results.

Because CRMP2 and endophilin2 coordinated with each other to promote synaptic AMPA receptor levels, we investigated the factors that could control the level of coordination between CRMP2 and endophilin2. Numerous studies have shown that phosphorylation is the most important posttranslational modification occurring in CRMP2. There are multiple phosphorylation sites at the C-terminal tail of CRMP2, such as Thr514/Ser518, Ser522, and Thr555, which can be phosphorylated by GSK3β, cyclin-dependent kinase 5 (Cdk5), and rho-associated protein kinase (RhoK), respectively (Arimura et al., 2005; Uchida et al., 2005; Yoshimura et al., 2005; Dustrude et al., 2016; Figure 5A). We used point mutation technology to mutate all the phosphorylation sites and construct a quadruple alanine mutant CRMP2^QmA^ (the four phosphorylation sites were mutated to alanine), which would mimic the dephosphorylated form of CRMP2, and a quadruple aspartic acid mutant CRMP2^QmD^ (the four phosphorylation sites were mutated to aspartic acid), which would mimic the phosphorylated form of CRMP2. First, we purified the fusion proteins of GST-CRMP2 and its mutants and semiquantitatively analyzed the proteins relative to the BSA standard (Figure 5B). Then, pull-down experiments were performed to analyze whether CRMP2 phosphorylation affected its binding to endophilin2. As shown in Figure 5C, the CRMP2 dephosphomimetic mutant (CRMP2^QmA^) had stronger affinity for endophilin2, whereas the CRMP2 phosphomimetic mutant (CRMP2^QmD^) only weakly bound to endophilin2, indicating that phosphorylation of CRMP2 changed its ability to bind to endophilin2. Moreover, because the binding of CRMP2 with tubulin had previously been reported to be phosphorylation dependent (Yoshimura et al., 2005), we performed pull-down assays to examine the relationship between CRMP2 and tubulin in a control trial. As shown in Figure 5D, the binding of CRMP2 to tubulin was phosphorylation dependent, as reported previously (Yoshimura et al., 2005). These results indicated that the phosphorylation of CRMP2 could decrease its interaction with endophilin2.

### Phosphorylation of CRMP2 Reduced the Surface Expression of GluA1 in Hippocampal Neurons

**Figure 6:**
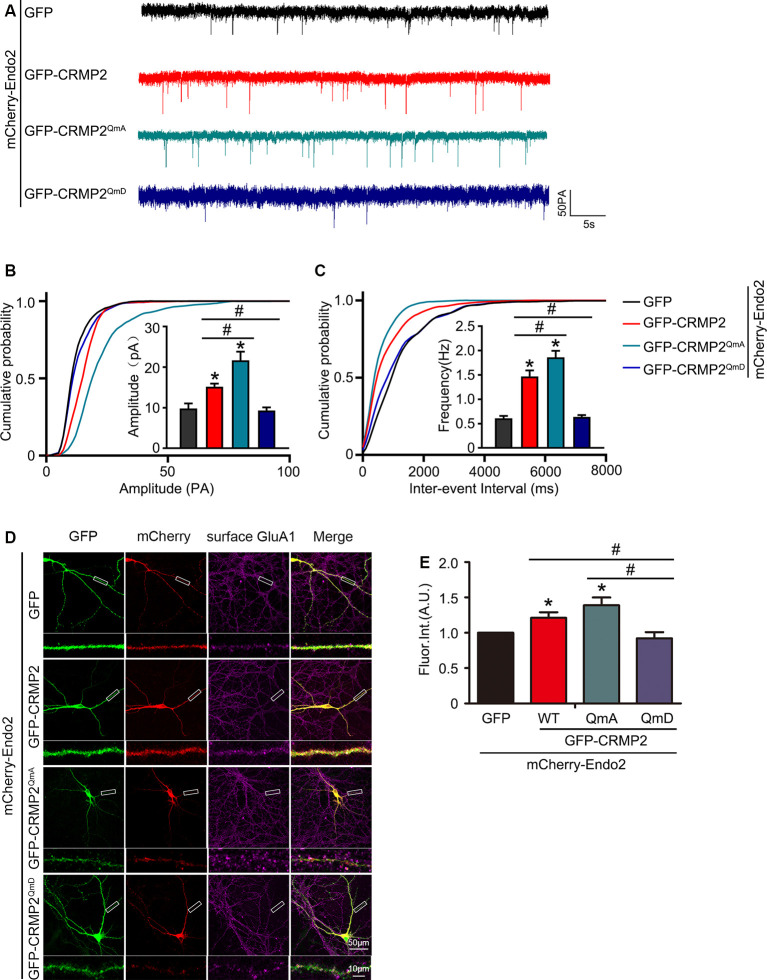
CRMP2 phosphorylation reduces the surface expression of GluA1 in hippocampal neurons. **(A)** Representative tracings of mEPSC recorded from cultured hippocampal neurons transfected with mCherry-Endo2 and GFP, mCherry-Endo2 and GFP-CRMP2, mCherry-Endo2 and GFP-CRMP2^QmA^, and mCherry-Endo2 and GFP-CRMP2^QmD^. **(B,C)** Cumulative probabilities of the mEPSC amplitude and interevent interval of neurons in these groups. Insets: histogram plots of mEPSC amplitude and frequency of neurons in these groups, *n* = 15 cells per group. *p < 0.05, as compared to the mCherry-Endo2 and GFP group, ^#^*p* < 0.05, as compared to the mCherry-Endo2 and GFP-CRMP2^QmD^ group. **(D)** Cultured hippocampal neurons transfected with mCherry-Endo2 and GFP, mCherry-Endo2 and GFP-CRMP2, mCherry-Endo2 and GFP-CRMP2^QmA^, and mCherry-Endo2 and GFP-CRMP2^QmD^ were immunostained with surface GluA1. Scale bar, 50 and 10 μm in the magnified dendrite. **(E)** Quantitative analysis of fluorescence intensity of surface GluA1 of neurons in these groups, *n* = 15 cells per group. *p < 0.05, as compared to the mCherry-Endo2 and GFP group, ^#^*p* < 0.05, as compared to the mCherry-Endo2 and GFP-CRMP2^QmD^ group.

Because the phosphorylation of CRMP2 prevented its interaction with endophilin2, we aimed to investigate whether CRMP2 phosphorylation affected synaptic AMPA receptor levels. To address this issue, CRMP2 and its different phosphorylation mutants were cotransfected with endophilin2 into DIV 12 hippocampal neurons. Then, 48 h after transfection, whole-cell patch clamp recordings were obtained for measurement of the mEPSC levels. As shown in Figures 6A–C, significant increases in mEPSC amplitude and frequency were observed in neurons cotransfected with CRMP2 and endophilin2, compared with those in neurons transfected with endophilin2 alone. Moreover, significant increases in mEPSC amplitude and frequency were also observed in neurons cotransfected with the CRMP2 dephosphomimetic mutant (CRMP2^QmA^) and endophilin2, compared with those in neurons transfected with endophilin2 alone or neurons cotransfected with CRMP2 and endophilin2. However, when neurons were cotransfected with the CRMP2 phosphomimetic mutant (CRMP2^QmD^) and endophilin2, the amplitude and frequency of mEPSCs decreased significantly, compared with those in neurons cotransfected with CRMP2 and endophilin2 or neurons cotransfected with the CRMP2 dephosphomimetic mutant (CRMP2^QmA^) and endophilin2. These results indicated that CRMP2 promoted the functions of synaptic AMPARs and that the dephosphorylation of CRMP2 further enhanced synaptic AMPA receptor levels. However, the phosphorylation of CRMP2 decreased the function of synaptic AMPARs. To obtain further clarity, we also performed immunofluorescence staining in nonpermeabilized neurons in order to detect surface GluA1 expression. As shown in Figures 6D,E, the fluorescence intensity of surface GluA1 in neurons cotransfected with CRMP2 and endophilin2 was significantly higher than that in neurons transfected with endophilin2 alone. Additionally, the fluorescence intensity of surface GluA1 in neurons cotransfected with the CRMP2 dephosphomimetic mutant (CRMP2^QmA^) and endophilin2 was also significantly higher than that in neurons transfected with endophilin2 alone and neurons cotransfected with CRMP2 and endophilin2. However, when neurons were cotransfected with the CRMP2 phosphomimetic mutant (CRMP2^QmD^) and endophilin2, the fluorescence intensity of surface GluA1 decreased significantly compared with that in neurons cotransfected with CRMP2 and endophilin2 or neurons cotransfected with the CRMP2 dephosphomimetic mutant (CRMP2^QmA^) and endophilin2. Taken together, our results demonstrated that dephosphorylation of CRMP2 increased the affinity of CRMP2 for endophilin2 and increased the surface expression of the GluA1 subunit, whereas inhibition of the interaction between phosphorylated CRMP2 and endophilin2 could induce the downregulation of GluA1 subunit surface expression.

### Phosphorylation of CRMP2 at Thr514/Ser518 Inhibited the Recycling of the AMPAR GluA1 Subunit *via* its Dissociation From Endophilin2

**Figure 7:**
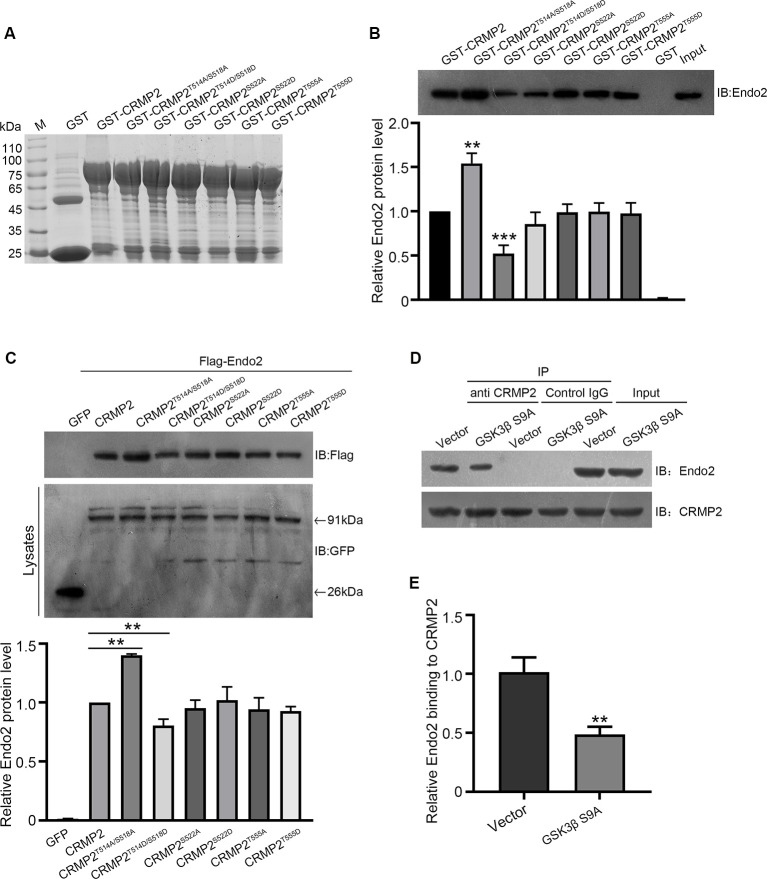
The phosphorylation of CRMP2 at Thr514/Ser518 reduces its interaction with endophilin2. **(A)** The expression and purification of glutathione-S-transferase (GST)-CRMP2 and its phosphorylated and dephosphorylated mutants. **(B)** Pull-down assays were conducted with GST-CRMP2 and its phosphorylated and dephosphorylated mutants in brain lysates of 1-month-old rats. Western blotting was performed with an anti-endophilin2 antibody (up); quantitative analysis of the relative binding of Endo2 to CRMP2 was performed (down), *n* = 3, **p < 0.01, ***p < 0.001 compared to GST-CRMP2 group. **(C)** HEK293 cells were cotransfected with Flag-Endo2 and GFP, GFP-CRMP2, and its dephosphomimetic and dephosphomimetic mutants, after which the co-IP assay was performed. Protein complexes that were coeluted with the anti-GFP antibody were detected using the anti-Flag antibody (up); quantitative analysis of the relative binding of Endo2 to CRMP2 was performed (down), *n* = 3, **p < 0.01 compared to the CRMP2 group. **(D)** Cultured hippocampal neurons were infected with Ad-HA vector and Ad-HA-GSK3β S9A (constitutively active GSK3β mutant containing a serine-to-alanine substitution at residue 9) for 24 h. The cell lysates were immunoprecipitated with anti-CRMP2 or the control immunoglobulin G (IgG) antibody, followed by the immunoblotting of immunoprecipitated samples with anti-endophilin2 antibody. **(E)** Quantification of Endo2-immureactive bands after normalization with coprecipitated CRMP2 levels, *n* = 3, **p < 0.01.

**Figure 8:**
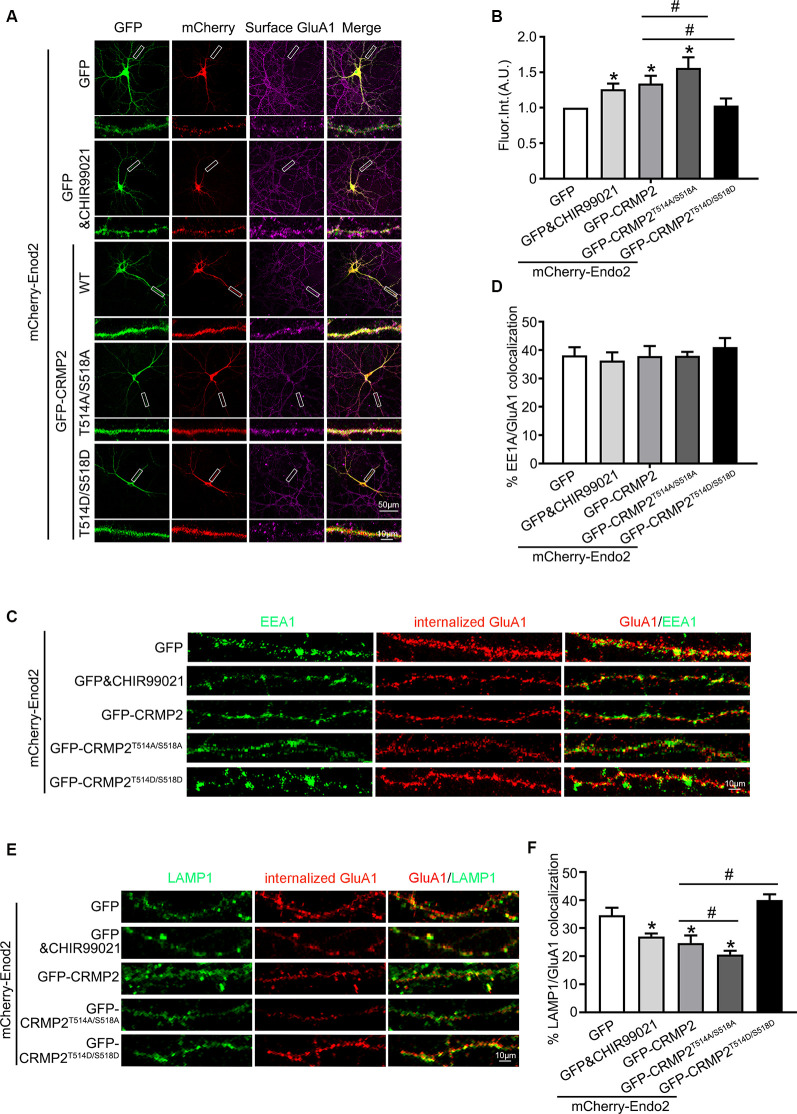
The phosphorylation of CRMP2 at Thr514/Ser518 reduces the surface expression and increases degradation of GluA1. **(A)** Cultured hippocampal neurons transfected with mCherry-Endo2 and GFP, and mCherry-Endo2 and GFP (incubated with CHIR99021), mCherry-Endo2 and GFP-CRMP2, mCherry-Endo2 and GFP-CRMP2^T514A/S518A^, and mCherry-Endo2 and GFP-CRMP2^T514D/S518D^; then, they were immunostained with surface GluA1. Scale bar, 50 and 10 μm in the magnified dendrite. **(B)** Quantitative analysis of fluorescence intensity of surface GluA1 of neurons in these groups, *n* = 15 cells per group, *p < 0.05, as compared to the mCherry-Endo2 and GFP group; ^#^*p* < 0.05, as compared to the mCherry-Endo2 and GFP-CRMP2 group. **(C,D)** Representative images **(C)** and quantitative analysis **(D)** show the colocalization of EEA1 and internalized GluA1 in hippocampal neurons. Neurons were coimmunostained at DIV12, using antibodies against GluA1 and EEA1. Scale bar, 10 μm. **(E,F)** Representative images **(E)** and quantitative analysis **(F)** show the colocalization of LAMP1 and internalized GluA1 in hippocampal neurons. Neurons were coimmunostained at DIV12, using antibodies against GluA1 and LAMP1. Scale bar, 10 μm. *n* = 15 cells per group. *p < 0.05, as compared to the mCherry-Endo2 and GFP group; ^#^*p* < 0.05, as compared to the mCherry-Endo2 and GFP-CRMP2 group.

After establishing the role of CRMP2 phosphorylation in AMPA receptor trafficking, we attempted to determine which phosphorylation site played a key role during this process. To investigate the specific phosphorylation site, we generated a series of mutations at residues Thr514/Ser518, Ser522, and Thr555 (replaced by alanine or aspartic acid), to prevent or mimic phosphorylation on these sites, in accordance with their corresponding kinases (Figure 5A). The phosphomimetic mutant or dephosphomimetic mutant forms of wild-type CRMP2 were expressed as GST-fusion proteins (Figure 7A). First, GST pull-down was performed, to examine the relationship between these CRMP2 constructs and endophilin2. In the pull-down experiment, equal amounts of GST or GST fusion proteins (~10 μg) were mixed with ~400 μg rat brain lysate and incubated. The results were evaluated by Western blotting, using anti-endophilin2 antibodies. Notably, the relative binding of endophilin2 to CRMP2^T514A/S518A^ increased significantly, whereas the relative binding of endophilin2 to CRMP2^T514D/S518D^ decreased markedly, compared with that of the relative binding of endophilin2 to wild-type CRMP2. However, other mutants of CRMP2 did not alter the ability of CRMP2 to bind to endophilin2 (Figure 7B). Next, we performed coimmunoprecipitation experiments to further clarify this issue. As shown in Figure 7C, interaction between CRMP2^T514A/S518A^ and endophilin2 was enhanced, whereas the binding of CRMP2^T514D/S518D^ to endophilin2 was markedly reduced. Simultaneously, quantitative analysis results also showed that there were no significant differences in the relative levels of binding of endophilin2 to CRMP2 among wild-type CRMP2, CRMP2^T522A^, CRMP2^T522D^, CRMP2^T555A^, and CRMP2^T555D^. These results suggested that the interactions between CRMP2 and endophilin2 were dependent on Thr514/Ser518 and were not related to the phosphorylation of Ser522 and Thr555. Because Thr514 and Ser518 were phosphorylated by GSK3β, we then tested whether GSK3β mediated CRMP2 phosphorylation to affect its ability to bind to endophilin2 in cells. Briefly, cultured hippocampal neurons were infected with Ad-HA and Ad-HA-GSK3β S9A (constitutively active GSK3β mutant containing a serine-to-alanine substitution at residue 9) for 24 h, and immunoprecipitation experiments were performed 3 days later. The results showed that the relative level of endophilin2 binding to CRMP2 decreased significantly in the GSK3β S9A group, compared with that in the control group (Figures 7D,E). Taken together, the above results indicated that CRMP2 phosphorylation at Thr514/Ser518 by GSK3β inhibited the binding of CRMP2 to endophilin2. To further determine the functional significance of the interaction between CRMP2 and endophilin2 in the regulation of AMPAR trafficking, surface and internalized levels of endogenous GluA1 were examined in cultured hippocampal neurons. We first transfected endophilin2 with CRMP2, CRMP2^T514A/S518A^, or CRMP2^T514D/S518D^ in cultured hippocampal neurons; then, 48 h after transfection, immunofluorescence staining was performed in nonpermeabilized neurons to detect the expression of surface GluA1. As shown in Figures 8A,B, the fluorescence intensity of surface GluA1 in neurons treated with ChiR99021, an inhibitor of GSK3β, and neurons cotransfected with CRMP2 and endophilin2 were significantly higher than that in neurons transfected with endophilin2 alone. Simultaneously, the level of surface GluA1 in neurons cotransfected with CRMP2^T514A/S518A^ and endophilin2 was significantly higher than that in neurons transfected with CRMP2 and endophilin2, as shown in Figure 6. However, the fluorescence intensity of surface GluA1 in neurons cotransfected with CRMP2^T514D/S518D^ and endophilin2 decreased significantly compared with that in neurons cotransfected with CRMP2 and endophilin2 or neurons cotransfected with CRMP2^T514A/S518A^ and endophilin2. We further labeled the internalized GluA1 in living cells after incubating cells with NMDA for 5 min and systematically examined their colocalization with early and late endosomes using the well-known markers EEA1 and LAMP1 (Chiu et al., 2017; Koszegi et al., 2017). We found that there were no differences among the five groups with regard to the colocalization of GluA1 with EEA1 (Figures 8C,D). However, the colocalization of GluA1 with LAMP1 in neurons treated with ChiR99021 or cotransfected with CRMP2 and endophilin2 was significantly lower than that in neurons transfected with endophilin2 alone, indicating that the level of degradation AMPAR was decreased. Simultaneously, the colocalization of GluA1 with LAMP1 in neurons cotransfected with CRMP2^T514A/S518A^ and endophilin2 was significantly lower than that in neurons transfected with CRMP2 and endophilin2. In addition, the colocalization of GluA1 with LAMP1 in neurons cotransfected with CRMP2^T514D/S518D^ and endophilin2 increased significantly, compared with that of neurons cotransfected with CRMP2 and endophilin2 or neurons cotransfected with CRMP2^T514A/S518A^ and endophilin2 (Figures 8E,F). These data suggested that the phosphorylation of CRMP2 at Thr514/Ser518 by GSK3β would increase the degradation of internalized GluA1 *via* its dissociation from endophilin2, thereby resulting in decreased surface expression of GluA1. However, the dephosphorylation of CRMP2 at Thr514/Ser518 would decrease the degradation of internalized GluA1 *via* its binding to endophilin2, resulting in increased surface expression of GluA1.

## Discussion

In this study, we demonstrated that CRMP2 coordinated with endophilin2 to regulate the recycling of the AMPA receptor GluA1 subunit. We first confirmed that CRMP2 physically interacted with the SH3 domain of endophilin2 through its C-terminus. Neurons with CRMP2 and endophilin2 overexpression exhibited increased GluA1 surface levels. Then, we verified that when their interaction was inhibited, the CRMP2 and endophilin2 overexpression phenotype failed to occur. Next, we found that the interaction between CRMP2 and endophilin2 was regulated by the phosphorylation of CRMP2. Finally, we observed that only the phosphorylation of CRMP2 at Thr514/Ser518 by GSK3β inhibited their interaction and the subsequent recycling of the AMPAR GluA1 subunit.

One of the most striking results from our study was that CRMP2 physically interacted with endophilin2 (Figures 1, 2). As reported previously, although high levels of both CRMP2 and endophilin2 are expressed in the central nervous system (Yoshimura et al., 2005; Chowdhury et al., 2006), their expression is not thought to be related. The results of bioinformatics analysis proved that these proteins may interact with each other (1A,B). Furthermore, pull-down and coimmunoprecipitation experiments confirmed that CRMP2 and endophilin2 interacted with each other *in vitro* (Figures 1C–F), and immunostaining results showed that endogenous CRMP2 and endophilin2 were colocalized in cell bodies and along the neurites. Additionally, our results confirmed that CRMP2 was bound to the SH3 domain of endophilin2 through its C-terminus (Figure 2). In previous studies, endophilin2 has been shown to colocalize with PSD95, suggesting that this protein may be abundantly expressed in postsynaptic regions (Chowdhury et al., 2006; Zhang et al., 2017). Moreover, endophilin2 has also been shown colocalize with GluA1, thereby regulating AMPAR trafficking (Zhang et al., 2017). The interaction of CRMP2 with endophilin2 suggested that these proteins may play key roles in the regulation of AMPAR trafficking.

Our results also provided direct evidence that the interaction between CRMP2 and endophilin2 was regulated by CRMP2 phosphorylation. Phosphorylation is the most important posttranslational modification of CRMP2 (Dustrude et al., 2016), and CRMP2 phosphorylation can change the ability of CRMP2 to bind to its interaction partners (Yoshimura et al., 2005). For example, CRMP2 binds to the tubulin heterodimer, whereas the phosphorylation of CRMP2 by GSK3β, RhoK, and Cdk5 lowers the binding affinity of CRMP2 to tubulin (Arimura et al., 2005; Uchida et al., 2005; Yoshimura et al., 2005; Khanna et al., 2012). In this study, we found that the CRMP2 dephosphomimetic mutant (CRMP2^QmA^) had a stronger affinity to endophilin2, whereas the CRMP2 phosphomimetic mutant (CRMP2^QmD^) was only weakly bound to endophilin2, suggesting that the phosphorylation of CRMP2 altered its ability to bind to endophilin2 (Figure 5). Moreover, the interaction between CRMP2^T514A/S518A^ and endophilin2 was enhanced, whereas the binding of CRMP2^T514D/S518D^ to endophilin2 was markedly reduced (Figures 7B,C). Additionally, the positive control tubulin also showed a phosphorylation-dependent ability to bind to CRMP2, as reported previously (Yoshimura et al., 2005; Figure 5D). Mutation at Ser522 or Thr555 of CRMP2 did not alter the ability of CRMP2 to bind to endophilin2 (Figures 7B,C) indicating that the interaction between CRMP2 and endophilin2 was dependent on GSK3β-mediated phosphorylation.

The results of the current study provided important insights into the mechanism through which CRMP2 cooperated with endophilin2 to regulate AMPAR trafficking. In this study, the amplitude and frequency of mEPSCs increased significantly in neurons transfected with CRMP2 and endophilin2 but decreased significantly when the interaction between CRMP2 and endophilin2 was disrupted by deletion of the SH3 domain of endophilin2 or the C-terminus of CRMP2 (Figure 3). The altered amplitude of mEPSCs may result from changes in the number of AMPARs at synapses. Furthermore, immunostaining of surface GluA1 in hippocampal neurons confirmed this phenomenon (Figure 4). Overall, these results suggested that the interaction of CRMP2 with endophilin2 was conductive to promote synaptic AMPAR levels. Because CRMP2 phosphorylation can alter the affinity between CRMP2 and endophilin2, when cultured neurons were cotransfected with the CRMP2 dephosphomimetic mutant and endophilin2, both the amplitude and frequency of mEPSCs were increased. In contrast, when neurons were cotransfected with the CRMP2 phosphomimetic mutant and endophilin2, the amplitude and frequency of mEPSCs were decreased (Figure 6). The same phenomenon was observed in immunostaining of surface GluA1 (Figures 6, 8). We concluded that CRMP2 had the ability to interact with endophilin2 and transfer AMPARs to the membrane; moreover, when CRMP2 was phosphorylated, CRMP2 and endophilin2 dissociated, and AMPAR could not be transferred to the membrane.

Dynamic AMPAR trafficking involves clathrin-dependent endocytosis, recycling, and exocytosis pathways (Shepherd and Huganirl, 2007; van der Sluijs and Hoogenraad, 2011; Parkinson and Hanley, 2018; Troyano-Rodriguez et al., 2019). Previous studies have shown that endophilin2 is involved in activity-dependent AMPAR endocytosis (Chowdhury et al., 2006; Zhang et al., 2017). We verified that endophilin2 interacted with GluA1 to mediate AMPAR endocytosis. However, the localization of AMPARs after internalization was not clear. The binding of CRMP2 with endophilin2 suggested the likelihood of involvement of CRMP2 in AMPAR trafficking. We found that cotransfection of the CRMP2 phosphomimetic mutant (CRMP2^T514D/S518D^) with endophilin2 caused aggregation of late endosomes, without inducing changes in the behavior of early endosomes. Cotransfection of the CRMP2 dephosphomimetic mutant (CRMP2^T514A/S518A^) with endophilin2 caused a decrease in late endosome levels, without any changes in early endosome levels (Figure 8). These findings implied that CRMP2 phosphorylation caused loss of binding ability with endophilin2, thereby resulting in increased degradation of internalized GluA1. The increased ability of CRMP2 to bind to endophilin2 induced a decrease in degradation and increase in the surface expression of GluA1 subunits. The binding of CRMP2 with endophilin2 may therefore serve as a bridge to regulate the recycling of AMPAR and determine synaptic strength and plasticity. In addition to mediating the functional plasticity of synapses, CRMP2 is known to enable their structural plasticity (Hensley et al., 2011; Abe et al., 2018). Indeed, CRMP2 and dephosphorylated CRMP2 cause immature dendritic spines to become mature mushroom-shaped dendritic spines, whereas phosphorylated CRMP2 has the opposite effect (Zhang et al., 2018). CRMP2 can bind to tubulin heterodimers to promote microtubule assembly, and actin and tubulin cytoskeletons are responsible for the formation and maturation of dendritic spines. Hence, the contribution of CRMP2 to synaptic plasticity is often attributed to its role in the cytoskeleton. The results of our current study showed that CRMP2 performed crucial roles in synaptic AMPAR delivery (Abe et al., 2018; Takahashi, 2019), which indicates the presence of another new mechanism for the maintenance of dendritic spine plasticity.

In conclusion, we identified a unique interaction between CRMP2 and the endocytotic protein endophilin2 and showed that their phosphorylation-regulated interaction was required for the recycling of GluA1-containing AMPARs. We also demonstrated that when CRMP2 was phosphorylated by GSK3β, it was not bound to endophilin2. Thus, the resulting recycled endosomes could not be transferred along the cytoskeleton, to the postsynaptic membrane, and the expression of surface AMPARs was decreased, which may cause synaptic inhibition. When CRMP2 was dephosphorylated, the binding affinity of CRMP2 and endophilin2 was increased. The combination of CRMP2 and endophilin2 enabled transfer of the recycling endosome along the cytoskeleton to the postsynaptic membrane. Therefore, the number of postsynaptic membrane AMPAR was increased, which then enhanced synaptic functioning. Our data provide new and important insights into the role of microtubule-associated and endocytotic proteins, which coordinate the recycling of AMPAR. Furthermore, these findings have improved our understanding of the mechanisms for AMPAR trafficking.

## Data Availability Statement

The datasets generated for this study are available on request to the corresponding author.

## Ethics Statement

The animal study was reviewed and approved by Institutional Animal Care and Use Committee at Jinan University.

## Author Contributions

CC and GG conceived and designed the study. JZ, JL, and YY performed most of the experiments and analyzed the data. XL and YJ performed the Western blotting. YW performed the simulation of protein binding confirmation. JZ wrote the article. CC and GG reviewed and edited the manuscript. All authors contributed to the article and approved the submitted version.

## Conflict of Interest

The authors declare that the research was conducted in the absence of any commercial or financial relationships that could be construed as a potential conflict of interest.

## Footnotes

^1^www.rcsb.org
